# Comment on Tauriainen et al.: Serum, liver and bile sitosterol and sitostanol in obese patients with and without NAFLD

**DOI:** 10.1042/BSR20180505

**Published:** 2018-10-31

**Authors:** Jogchum Plat, Sabine Baumgartner, Tom Houben, Anita C.E. Vreugdenhil, Ronald P. Mensink, Dieter Lütjohann, Ronit Shiri-Sverdlov

**Affiliations:** 1 Department of Nutrition and Movement Sciences, School for Nutrition and Translational Research in Metabolism (NUTRIM), Maastricht University, Maastricht, The Netherlands; 2 Department of Molecular Genetics, School for Nutrition and Translational Research in Metabolism (NUTRIM), Maastricht University, Maastricht, The Netherlands; 3 Department of Pediatrics, Maastricht University Medical Center, Maastricht, The Netherlands; 4 Institute of Clinical Chemistry and Clinical Pharmacology, Bonn University, Bonn, Germany

**Keywords:** nutrition, nash, non alcoholic fatty liver disease, plant sterols

## Abstract

This short article provides a comment on the recent article by Tauriainen et al. [*Bioscience Reports* (2018) **38**, BSR20171274 https://doi.org/10.1042/BSR20171274]

In a previous issue of *Bioscience Reports*, Tauriainen et al. [1] present data on the relations between serum, liver, and bile concentrations of sitosterol, campesterol, and sitostanol standardized for cholesterol in morbidly obese subjects with a normal liver or a fatty liver further subdivided into simple steatosis or NASH. In our opinion this is the first time that these non-cholesterol sterols have been reported in human liver samples – especially in conditions of a fatty liver compared with control – which is therefore of great scientific value. In fact, it is highly relevant to understand the associations and potential causalities regarding hepatic non-cholesterol sterols and liver health. The fascinating question is why on one side dietary supplementation of both plant sterol as well as plant stanol esters seems to protect against liver inflammation in animal models for NASH [2,3] whereas at the same time soy-based parenteral nutrition which is rich in plant sterols results in cholestasis, elevated transaminases and bilirubin levels, and liver damage [4,5]. To be able to understand these effects, there is a clear need for this type of fundamental data regarding differences in plant sterol and stanol concentrations in disease conditions, but also with respect to the potential effects of changing non-cholesterol sterol concentrations in that particular disease.

However, in the paper by Tauriainen et al. [1] there are some methodological issues that raise concerns, which we feel need to be addressed. *First*, while concentrations of three non-cholesterol sterols were reported, measurements of others like desmosterol, lathosterol, stigmasterol, and campestanol are missing. Especially it is important to measure desmosterol, as it was earlier described as a marker to differentiate between NAFLD and NASH [6], which our group later confirmed [2]. *Second*, the fact that in this cross-sectional study without supplying any dietary plant sterols or stanols, there were opposite effects of sitosterol and sitostanol, should certainly be explained. Since the physiology of plant sterols and stanols is very comparable sharing the same intestinal and hepatic transporters for uptake and secretion, it is in our opinion difficult to describe a biological plausible mechanism by which sitosterol would show a negative association with hepatic inflammation, whereas sitostanol would show the opposite. *Third*, the authors concluded that hepatic sitosterol concentrations correlate negatively with steatosis and lobular inflammation, while sitostanol concentrations show a positive correlation with liver histology markers. However, we question whether these conclusions are correct since they are based on Spearman correlations (Table 2 in the original paper [1]). To our opinion, it is not appropriate to perform this analysis between continuous (plant sterol and stanol concentrations) and discrete variables (liver histology). *Fourth*, while most of the non-cholesterol sterol data are listed in relation to cholesterol levels, other data are more difficult to find as they are presented mostly as correlations and not given as absolute values.

*Fifth* and last, probably most important, we wonder whether the remarkable observation regarding sitostanol is related to the fact that sitostanol concentrations were measured by GC-FID using 5α-cholestane as the internal standard. By definition, an internal standard should have similar chemical properties as the compound to be calculated, and should additionally be added in similar amounts as the compound to be quantitated. Here cholesterol, plant sterols, and plant stanols are quantitated using 5α-cholestane as the internal standard, which does not at all fulfil these preconditions for an appropriate internal standard for all sterols measured. The levels of cholesterol, plant sterols and stanols differ in a range of 10^4^. Moreover, it is known that 5α-cholestane is lacking the hydroxyl group at C3 and may therefore potentially behave differently with cholesterol and non-cholesterol sterols during sample pre-treatment. The fact that hepatic sitostanol was measured by GC-FID also introduces the most confounding methodological issue since it is well known that it is challenging to measure very low concentrations of plant stanols by GC-FID due to the likely impact of co-eluting compounds. This is already a problem in plasma samples but will be especially sensitive in hepatic tissues where plant stanol concentrations may even be lower as compared with serum. The actual problem is that the co-eluting compounds are often cholesterol precursors, which in hepatic samples are even higher as compared with plasma samples. This is not a real problem for plant sterols, which co-elute at other retention times. Therefore, it is very questionable whether the hepatic sitostanol peak is not predominantly a cholesterol precursor signal, especially since earlier observations showed that desmosterol was elevated in NASH livers [6]. Moreover, the presence of a correlation between plasma and hepatic sitosterol concentrations, while at the same time there is a lack of correlation between plasma and hepatic sitostanol concentrations is remarkable. In our hands, plasma and tissue concentrations for plant sterols as well as for stanols always shows a high correlation for all tissues analyzed, so also for liver, at least when measured via GC-MS. Therefore, the fact that Tauriainen et al. [1] do find a correlation between hepatic and plasma sitosterol concentrations but not for sitostanol is again suggestive for a potential problem in their analysis of hepatic sitostanol via GC-FID.

Finally, we want to position a clear plead to perform placebo-controlled intervention studies to evaluate whether plant sterols and stanols affect liver health or not instead of relying on these type of association studies. In this context the recent paper of Javanmardi et al. [7] shows potential beneficial effects of a plant sterol enriched diet.

To conclude, here we suggest the possibility that the observed finding by Tauriainen et al. [1] regarding the positive association between sitostanol and lobular inflammation is likely to be a consequence of the applied analytical methodology.
